# Laparoscopic Fixation of the Proximal Jejunum and Gastrojejunostomy for Small Bowel Volvulus and Recurrent Duodenal Obstruction Caused by Incomplete Fixation and Abnormal Mobility of the Ligament of Treitz: A Case Report

**DOI:** 10.70352/scrj.cr.26-0256

**Published:** 2026-06-17

**Authors:** Tomoyuki Fukami, Hiroshi Minato, Toshiyuki Kosuga, Tomohiro Arita, Atsushi Shiozaki

**Affiliations:** 1 Division of Digestive Surgery, Department of Surgery, Yujin-Yamazaki Hospital, Hikone, Shiga, Japan; 2 Division of Digestive Surgery, Department of Surgery, Kyoto Prefectural University of Medicine, Kyoto, Kyoto, Japan

**Keywords:** ligament of Treitz, duodenojejunal junction, laparoscopic surgery

## Abstract

**INTRODUCTION:**

The ligament of Treitz plays a critical role in maintaining the normal anatomical position of the duodenojejunal junction by suspending and fixing the proximal jejunum to the retroperitoneum.

**CASE PRESENTATION:**

A 77-year-old man receiving home-visit medical care after cerebral infarction and cerebral hemorrhage, who had undergone tracheostomy and gastrostomy, was admitted for aspiration pneumonia with respiratory failure. An abdominal CT demonstrated dilatation from the stomach to the proximal duodenum. The third portion of the duodenum coursed caudally without passing between the abdominal aorta and the superior mesenteric artery (SMA). The superior mesenteric vein was located to the right of the SMA at the L2 vertebral level, but to the left of the SMA at the L4 vertebral level, suggesting mesenteric rotation. After conservative decompression therapy, repeat imaging showed spontaneous restoration of normal anatomy. Given the patient’s history of recurrent aspiration pneumonia secondary to duodenal obstruction, elective laparoscopic surgery was performed. Intraoperatively, no intestinal malrotation or internal hernia was identified. The duodenojejunal junction showed incomplete retroperitoneal fixation, and the suspensory tissue corresponding to the ligament of Treitz was lax. Laparoscopic fixation of the proximal jejunum to the left retroperitoneum and gastrojejunostomy were performed. The patient was discharged on POD 41. No recurrent obstruction or aspiration events were observed during 9 months of follow-up.

**CONCLUSIONS:**

We report a rare case of recurrent duodenal obstruction with transient small bowel volvulus associated with incomplete fixation and abnormal mobility of the ligament of Treitz. When intermittent rightward displacement of the duodenojejunal junction is observed, this condition should be considered in the differential diagnosis in addition to intestinal malrotation.

## Abbreviations

AA
: abdominal aorta
SMA
: superior mesenteric artery
SMV
: superior mesenteric vein

## INTRODUCTION

The ligament of Treitz plays a critical role in maintaining the normal anatomical position of the duodenojejunal junction by suspending and fixing the proximal jejunum to the retroperitoneum. Abnormal mobility or incomplete fixation of the ligament of Treitz may cause intermittent obstruction and diagnostic confusion with intestinal malrotation. We report a rare case of recurrent duodenal obstruction with transient small bowel volvulus caused by incomplete fixation and abnormal mobility of the ligament of Treitz, which was successfully treated with laparoscopic fixation of the proximal jejunum and gastrojejunostomy.

## CASE PRESENTATION

A 77-year-old man receiving home-visit medical care after cerebral infarction and cerebral hemorrhage, who had undergone tracheostomy and gastrostomy, was admitted with respiratory failure and increased tracheostomy drainage and was diagnosed with aspiration pneumonia.

**Fig. 1:**
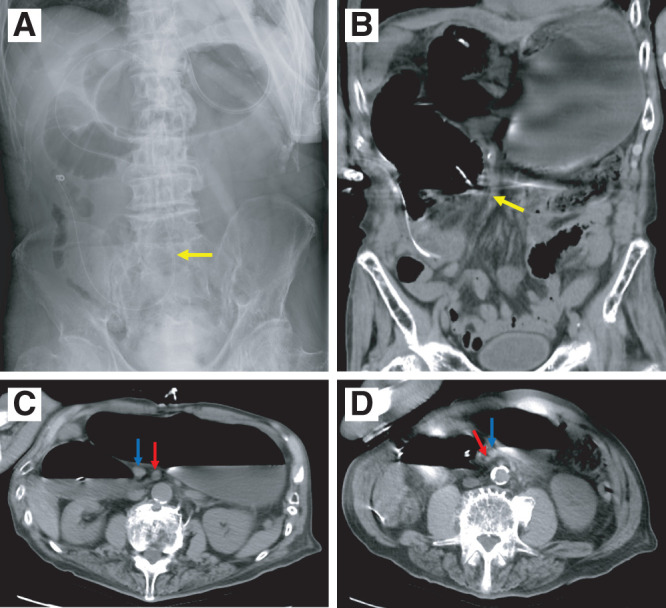
Imaging findings in small bowel volvulus. Anteroposterior abdominal X-ray (**A**) and abdominal multidetector CT images—coronal section (**B**), and axial sections at the L2 vertebral level (**C**) and L4 vertebral level (**D**). A percutaneous endoscopic gastrojejunostomy tube is in place. The third portion of the duodenum (yellow arrow) coursed caudally without passing between the AA and the SMA. At the L2 vertebral level, the SMV (blue arrow) was located to the right of the SMA (red arrow), whereas at the L4 vertebral level, the SMV was located to the left of the SMA. AA, abdominal aorta; SMA, superior mesenteric artery; SMV, superior mesenteric vein

On admission, blood pressure was 126/63 mmHg, pulse rate 65/min, body temperature 37.0°C, and peripheral oxygen saturation 93%. Height and weight were 158 cm and 51 kg, respectively. Physical examination showed no abnormal findings in the cervical or thoracic regions. The abdomen was flat and soft, without tenderness or guarding. Laboratory findings were as follows: white blood cell count 4200/µL, hemoglobin 11.2 g/dL, and C-reactive protein 4.36 mg/dL. Abdominal X-ray and abdominal multidetector CT revealed dilatation from the stomach to the proximal duodenum. The third portion of the duodenum coursed caudally without passing between the AA and the SMA (**Fig. 1A** and **1B**). At the L2 vertebral level, the SMV was located to the right of the SMA, whereas at the L4 vertebral level, it was located to the left (**Fig. 1C** and **1D**). No definite whirl sign was observed. No abnormality of colonic position or fixation was identified.

**Fig. 2:**
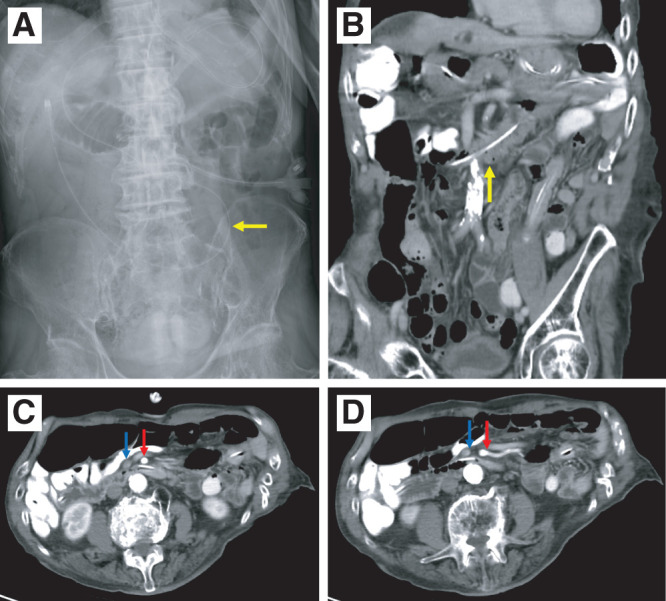
Imaging findings after spontaneous resolution of small bowel volvulus. Anteroposterior abdominal X-ray (**A**) and abdominal multidetector CT images—coronal section (**B**), and axial sections at the L2 vertebral level (**C**) and L4 vertebral level (**D**). A percutaneous endoscopic gastrojejunostomy tube is in place. The third portion of the duodenum (yellow arrow) traversed between the AA and the SMA from right to left, and the duodenojejunal junction had naturally relocated to the left abdomen. At both the L2 vertebral level and the L4 vertebral level, the SMV (blue arrow) was located to the right of the SMA (red arrow). AA, abdominal aorta; SMA, superior mesenteric artery; SMV, superior mesenteric vein

Intestinal malrotation was initially considered. However, after conservative decompression therapy, follow-up CT demonstrated spontaneous restoration of normal anatomy: the third portion of the duodenum traversed between the AA and SMA, and the duodenojejunal junction had naturally relocated to its typical position in the left abdomen (**Fig. 2A** and **2B**). Furthermore, the SMV restored its normal position to the right of the SMA at both vertebral levels (**Fig. 2C** and **2D**).

Over the previous 6 months, the patient had been hospitalized 3 times for aspiration pneumonia secondary to duodenal obstruction, and decompression via the gastrostomy tube had been performed on each occasion. Because similar episodes recurred repeatedly over a short period, elective laparoscopic surgery was performed during the present admission.

### Surgical findings and procedure

**Fig. 3:**
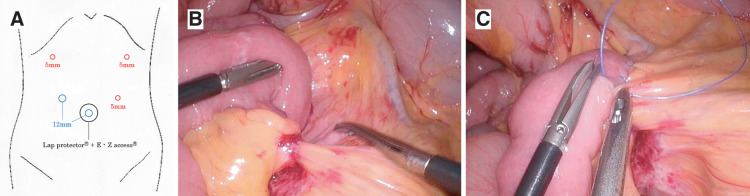
Laparoscopic port placement and intraoperative findings. (**A**) Port placement. (**B**) The structure corresponding to the ligament of Treitz was identified. The fixation of the duodenojejunal junction to the retroperitoneum was incomplete, and the suspensory fibromuscular tissue extending from the duodenal wall was lax. (**C**) The proximal jejunum was fixed to the left retroperitoneum using continuous suturing with a non-absorbable 3-0 barbed suture (V-Loc; Medtronic, Minneapolis, MN, USA).

A 30-mm skin incision was made at the umbilicus to perform laparotomy. An EZ Access device (Hakkko Medical, Tokyo, Japan) with a 12-mm port was placed on the Lap Protector (FF0707; Hakko Medical), and pneumoperitoneum was initiated. A 12-mm port was inserted at the lateral edge of the right rectus abdominis muscle, and 5-mm ports were placed in the bilateral subcostal regions and at the lateral edge of the left rectus abdominis muscle, for a total of 5 ports (**Fig. 3A**).

The duodenojejunal junction was inspected. No intestinal malrotation, adhesive band, or paraduodenal hernia was present. The colon showed normal position and fixation. The structure corresponding to the ligament of Treitz was identified. However, fixation of the duodenojejunal junction to the retroperitoneum was incomplete, and the suspensory fibromuscular tissue extending from the duodenal wall was lax (**Fig. 3B**).

The proximal jejunum adjacent to the duodenojejunal junction was fixed to the left retroperitoneum without tension using continuous seromuscular bites with a nonabsorbable 3-0 barbed suture (V-Loc; Medtronic, Minneapolis, MN, USA) over a length of approximately 5 cm, with care taken to avoid injury to mesenteric vessels and retroperitoneal structures (**Fig. 3C**).

In addition, the jejunum approximately 20 cm distal to the ligament of Treitz was elevated through an antecolic route, and gastrojejunostomy was performed. A Braun anastomosis was added 10 cm distal to the gastrojejunostomy. The mesenteric defect was closed with 3-0 barbed suture (V-Loc) to prevent internal hernia.

The postoperative course was uneventful. The patient was discharged on POD 41. During the 9-month postoperative follow-up, neither recurrent bowel obstruction nor aspiration occurred, and enteral nutrition was stable.

## DISCUSSION

The ligament of Treitz consists of fibromuscular tissue connecting the duodenojejunal junction to the retroperitoneum and contributes to the positional stability of the proximal jejunum. The ligament of Treitz consists of 2 parts. The superior part, referred to as the Hilfsmuskel, extends from the right diaphragmatic crus to the region surrounding the celiac artery. The inferior part suspends the third and fourth portions of the duodenum and the duodenojejunal junction from the retroperitoneum, thereby providing fixation.^1)^ Unlike other abdominal ligaments, the ligament of Treitz has not been identified on imaging modalities such as CT or MRI.^2)^

The principal differential diagnosis in this case was intestinal malrotation. Intestinal malrotation commonly involves congenital abnormal rotation and fixation of the midgut, often with persistent malposition of the duodenojejunal junction.^3,4)^ In the present case, CT examination at admission revealed an abnormal positioning of the duodenojejunal junction and dilatation extending from the stomach to the second portion of the duodenum, which was considered to correspond to the incomplete rotation type in Nishijima’s classification of intestinal malrotation.^5)^ Nevertheless, given that the duodenojejunal junction spontaneously returned to its normal anatomical position following preoperative conservative decompression therapy, this case was atypical for intestinal malrotation. SMA syndrome was also considered as a differential diagnosis; however, the primary abnormality was displacement of the duodenojejunal junction rather than persistent vascular compression of the third portion of the duodenum; therefore, this diagnosis was ruled out. Paraduodenal hernia was unlikely because no hernia sac or defect was identified intraoperatively. Adhesive kinking was also unlikely because the patient had no prior laparotomy and no adhesions were observed. Accordingly, transient rotational displacement associated with incomplete fixation and abnormal mobility of the duodenojejunal junction was considered the most plausible explanation.

The duodenum, the initial segment of the small bowel, is located predominantly within the anterior pararenal space and the retroperitoneum, except for its proximal segment, which is intraperitoneal.^2)^ Oyanagi et al. reported that when the duodenojejunal junction is displaced to the right, organs in the anterior pararenal space, including the pancreas, also shift to the right.^6)^ Although retroperitoneal organs, including the pancreas and duodenum, have conventionally been considered fixed and immobile structures, recent studies have reported pancreatic movement during respiration and positional changes. This mobility may be associated with age-related fragility of the retroperitoneal tissues.^7,8)^

**Fig. 4:**
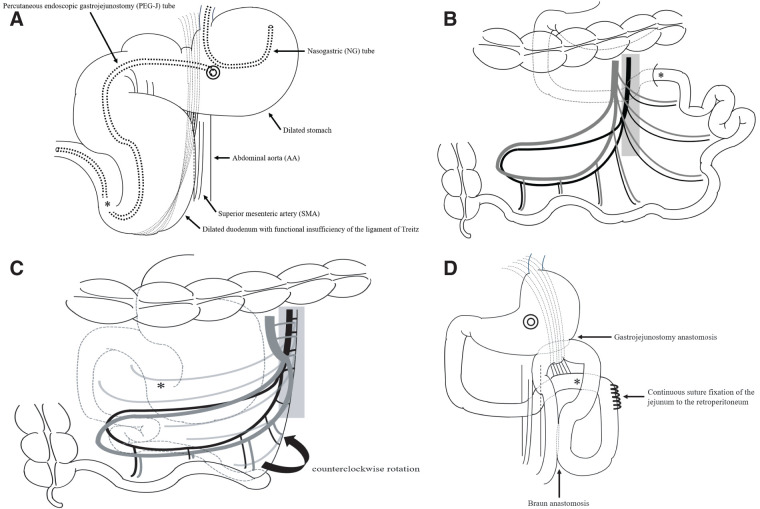
Schematic illustrations. (**A**) Duodenal obstruction secondary to small bowel volvulus inferred from CT images: anatomical relationships among the duodenum, duodenojejunal junction (*), and the ligament of Treitz. (**B**) Appearance of the duodenum, duodenojejunal junction (*), small bowel, and mesentery during small bowel volvulus, as inferred from CT images. (**C**) Appearance of the duodenum, duodenojejunal junction (*), small bowel, and mesentery after spontaneous resolution of the volvulus. (**D**) Postoperative configuration following laparoscopic fixation of the proximal jejunum and gastrojejunostomy.

In the present case, fixation of the duodenojejunal junction to the retroperitoneum was incomplete, and the suspensory fibromuscular tissue extending from the duodenal wall was lax (**Fig. 4A**).

The duodenojejunal junction temporarily returned to its normal anatomical position following preoperative decompression therapy (**Fig. 4B**), suggesting that the abnormal position was caused not by congenital malrotation but by excessive mobility resulting from inadequate retroperitoneal fixation. The recurrent duodenal obstruction was considered to result from counterclockwise rotation of the incompletely fixed duodenum and proximal jejunum around the SMA associated with positional changes, leading to displacement to the right side of the AA and subsequent duodenal kinking and stenosis (**Fig. 4C**).

Gastrojejunostomy was added in addition to proximal jejunum fixation because the patient had recurrent aspiration due to duodenal obstruction and was in a fragile general condition. A reliable bypass route was deemed necessary in case duodenal obstruction recurred even after fixation (**Fig. 4D**). This combined procedure resulted in a favorable postoperative course without recurrence.

This report has limitations. It describes a single patient with only 9 months of follow-up, and the exact mechanism cannot be definitively proven.

Reports describing surgical treatment for incomplete fixation and abnormal mobility of the ligament of Treitz without intestinal malrotation are extremely limited. To the best of our knowledge, based on a PubMed review, few laparoscopic cases have been described.^6)^ Therefore, the present report provides important insights into the pathophysiology and surgical management of incomplete fixation and abnormal mobility of the ligament of Treitz, suggesting that individualized laparoscopic fixation with adjunctive gastrojejunostomy can be effective in selected patients.

## CONCLUSIONS

Incomplete fixation and abnormal mobility of the ligament of Treitz can mimic intestinal malrotation and cause intermittent duodenal obstruction with transient mesenteric rotation. When temporary rightward displacement of the duodenojejunal junction that subsequently resolves spontaneously is observed, this condition should be considered in the differential diagnosis. Laparoscopic fixation of the proximal jejunum combined with selective gastrojejunostomy may be an effective treatment strategy in selected patients.

## DECLARATIONS

### Availability of data and materials

Data and materials are available from the corresponding author.
